# Comparative study of polyphenolic compound extraction from empty palm fruit bunches and sugarcane pulp

**DOI:** 10.1016/j.heliyon.2022.e08951

**Published:** 2022-02-11

**Authors:** Leonardo Satriono Putra, Johan Sukweenadhi, Clairine Nathania, Enrico Setiawan Wibowo, Gisela Buschle-Diller, Maria Goretti Marianti Purwanto

**Affiliations:** a Faculty of Biotechnology, University of Surabaya, Raya Kalirungkut, Surabaya 60293, Indonesia; b Department of Biosystems Engineering, Auburn University, AL 36849, USA

**Keywords:** Antioxidants, Empty palm fruit bunches, Extraction, Sugarcane pulp

## Abstract

Polyphenolic compounds have many benefits, one of which being their efficacy as antioxidants. They can be extracted from various parts of plants and from agricultural waste. In this research, sugarcane pulp, and empty palm fruit bunches from the palm oil production were investigated as potential raw materials. This study aims to determine solvents and easy-to-perform extraction methods that show the highest effectivity in regards to total phenolic and flavonoid yield and the correlated antioxidant activity. Extraction methods comprised maceration, Soxhlet extraction, and ultrasound assisted extraction (UAE); solvents that were investigated included water, 70% methanol and 70% ethanol. The antioxidant activity was measured by the DPPH (diphenyl-2-picrylhydrazyl) method and FRAP (Ferric Reduction Ability of Plasma) method. Based on the amount of polyphenol compounds as well as the antioxidant activity, the experiments showed that Soxhlet extraction with 70% methanol as solvent worked best for palm bunch waste and sugar cane pulp, resulted in about two times higher values for total phenolic content, flavonoid content and FRAP antioxidant activity as well as extract mass (yield) compared to the results from other extraction methods or solvents used in this experiment. The antioxidant activity of the extracts as measured by DPPH method seemed also to be promising, although the trend among solvent and extraction method was rather inconclusive.

## 1 Introduction

Indonesia is an agrarian country very rich in natural resources. Its climate and soil conditions are ideal for the cultivation of a large variety of plants that belong to important food staples. Examples of such plants include rice, palm oil, and sugarcane. Based on 2021 report from the Badan Pusat Statistik (BPS), sugar cane crop production reached 2,12 million tons in 2020. According to the Directorate General of Plantations, the Ministry of Agriculture and the Indonesian Palm Oil Entrepreneurs Association (GAPKI), the volume of palm oil crop production in Indonesia has continuously been growing and has already reached 51.63 million tons in 2020 (GAPKI, 2021). Increasing production, on the other hand, also leads to the accumulation of biowaste. It is not surprising that sugarcane pulp, and empty fruit bunches after palm oil harvest are building up in large quantities with every ton of food produced. Unfortunately, this biowaste is typically destroyed by burning in the fields, causing additional air pollution. Attempts have been made to transform biowaste into bioenergy or biofuel (Comelli et al., 2020; Ban et al., 2017) which, however, require well-functioning logistics and a relatively low moisture content of the residues to be economically feasible. Another common use for agricultural waste is in animal husbandry. Sugar cane bagasse, however, does not qualify as animal feed though due to the low digestibility (Molavian et al., 2020).

Instead of disposing of agricultural waste it could, however, serve as a valuable resource for polyphenolic compounds with antioxidant properties (Tsouko et al., 2019). Phenolic acids, flavonoids, and anthocyanins are produced by plants as a protective mechanism to counteract biotic and abiotic factors. The positive effect of phenolic compounds on human health is well-known (Cory et al., 2018). For instance, regular green tea consumption has proven to enhance the cognitive function in elderly persons (Cory et al., 2018; Singh et al., 2015). In addition to antioxidant properties they may also show anti-inflammatory effects, both in inflammation mediated by reactive oxygen species (ROS) and induced by cytokines (Colombo et al., 2019; Zhang and Tsao, 2016). High-value applications of polyphenolic compounds include admixtures to functional foods (Lapornik, 2005) as natural antioxidants and additional nutrients, potential treatment for neurodegenerative diseases (Lantz et al., 2020), additions to blood pressure-lowering drugs (Fraga et al., 2021), for antimicrobial activity (Rather et al., 2021) or in combination with the functionalization of nanoparticle for various physiological effects (Alomary & Ansari et al., 2021; Sati et al., 2020). They can also be admixed to animal feed (Shi et al., 2005) or serve as nontoxic antioxidants in active food packaging (Vilela et al., 2018).

Polyphenols can be isolated from agricultural waste through an extraction process using organic solvents, such as methanol and ethanol as well as by polar solvents such as water or apolar solvents such as hexane (Shi et al., 2005). The solvents for the extraction process are selected based on the polarity of the target metabolite. Extraction with aqueous solvents is generally performed to obtain polar compounds while organic solvents aim to separate compounds of lower polarity and low molecular weight, such as saponins and flavonoids. In addition to the choice of the solvent, the method of extraction plays an important role. Several methods, such as maceration (Vianney et al., 2018), ultrasound assisted extraction (UAE) (Filho et al., 2021; Tsouko et al., 2019), and Soxhlet or reflux extraction (Shi et al., 2021; Li et al., 2013; Han and May, 2012) can also be employed. In general, extraction condition, such as temperature and time plays a role on the stability of the extracted compounds, yield, and efficiency of the processes (Lameirao et al., 2020). The above report mostly focused on optimizing the condition for a single method and one type of agrowaste.

In this research, based on request from our industrial partner, two types of agricultural waste products that amass in Indonesia, namely sugar cane bagasse and empty fruit bunches of oil palms, have been investigated for the total phenolic content and its antioxidant capacity, using data from the existing reports from other researchers above. The easiest, yet most efficient extraction process in each case was sought, since the target community/industries in Indonesia include also small and medium one with limited budget and knowledge of sophisticated extraction techniques and skills. Readily available and pretty cheap solvents and equipment were selected for the extraction. Thus, this project could lay the foundation of the attempt to not only reduce the amount of biowaste but also to obtain valuable raw materials for functional and nutritional additives.

## 2 Materials and methods

### 2.1 Materials and equipment

The waste of empty palm fruit bunches was obtained from plantations in Sampit, Central Kalimantan, Indonesia. Sugarcane bagasse was acquired from Surabaya, East Java, Indonesia. Folin-Ciocalteu reagent, sodium carbonate anhydrous, gallic acid, sodium nitrite, aluminium chloride, sodium hydroxide, catechin, DPPH (diphenyl-2-picrylhydrazyl), methanol, ethanol, sodium acetate anhydrous, acetic acid, TPTZ (2,4,6-tripyridyl-s-triazine), hydrochloric acid, iron(III) chloride hexahydrate, and distilled water were used for the extraction and the analysis of the extracts. All chemicals were purchased from Merck (Indonesia), analytical grade and used without further purification.

For the determination of the polyphenolic and the flavonoid content of the samples, a UV-Vis spectrophotometer (Genesys 105 UV-Vis, Thermo Scientific, USA) and a microplate reader (FLUOstar Omega, BMG LabTech, USA) were used. Microplate reader was also employed for determination of antioxidant capacity with less sample volume.

### 2.2 Sample preparation

Samples for extraction were prepared by first thoroughly washing the agroindustrial waste (empty palm fruit bunches and sugarcane pulp) with distilled water, then cutting it into small pieces using a blender and drying it in an oven (Memmert GmbH, Germany) at 50 °C for three days or until it reached a constant weight. The fibers were sieved using a 40-mesh filter to yield a fibrous powder of uniform consistency.

### 2.3 Extraction methods

The goal of the project was to identify the most efficient solvent and the best, yet simplest extraction method for obtaining polyphenolic compounds from agricultural waste in considerable amounts so that the method could easily be upscaled for industrial purposes. If economically feasible processes can be developed, the extracted compounds might find many value-added applications as antioxidants in food, cosmetic and other industries. The success of the extraction will be measured by several common parameters related to the antioxidant capacity, i.e. Total Phenolic Content, Total Flavonoid Content and Antioxidant Activity.

Solvent extractions using three different methods were carried out with 3 different solvents, namely 70% aqueous ethanol, 70% aqueous methanol, and distilled water. The maceration method was basically chosen because it is simple and cheap. The other methods (Ultrasonic Assisted Extraction/UAE and Soxhlet Extraction) were carried out to expect less extraction time needed since the power used in UAE technique and higher temperature in Soxhlet Extraction may help to break the plant cell wall of empty palm fruit bunches and sugar cane pulp.

The maceration process was carried out by following the protocol of Vianney et al. (2018), in which 2.5 g of sample were immersed in 150 mL solvent and stirred for 72 h at 40 °C in a waterbath. Thereafter the solvent was evaporated with a rotary evaporator (Heidolph, Germany) and the extract was redissolved in the same solvent to 50 mL for analytical purposes (Lapornik et al., 2005).

UAE was carried out for 30 min at room temperature using an ultrasonicator (Branson Ultrasonic 1510, USA) with a frequency 40 kHz (Park et al., 2006). For this method, 2.5 g of sample were immersed in 150 mL solvent and after 30 min the solvent was removed by a rotary evaporator to determine the obtained extract mass. The extract was redissolved in the same solvent to 50 mL.

Soxhlet extraction was performed using 5 g of extract with 300 mL of solvent at a temperature of 60 °C for 2 h. After 2h the solvent was removed by a rotary evaporator to measure the extract mass and the extract was redissolved in the same solvent to 50 mL.

### 2.4 Determination of total phenolic content

Total phenolic content was measured using Folin-Ciocalteu method. The working protocol was based on Vianney et al. (2018) with slight modification, namely 0.1 mL extract was mixed with 0.2 mL of Folin-Ciocalteu reagent, 2 mL distilled water, and 1 mL of 15% anhydrous sodium carbonate (w/v). The solution was then stored in a dark room for 1 h and the absorbance determined with a UV-Vis spectrophotometer at a wavelength of 765 nm, using gallic acid as standard. Blank solutions were prepared by the same method, replacing the sample solution with distilled water. Three replications of the test were performed. The total phenolic content was calculated by Eq. (1)

(1) $$\text{Total ​phenolic ​content}=\frac{\text{ ​Gallic ​acid ​equivalent ​}\left(\text{GAE}\right)\left(\text{mg}\right)}{\text{ ​g ​dry ​mass ​sample}}$$

### 2.5 Total flavonoid content

Total flavonoid content assay was conducted based on the research of Benariba et al. (2013) with modifications, namely 0.1 mL extract (dissolved in the respective solvent) was mixed with 0.15 mL 15% aqueous NaNO_2_ and 0.15 mL 10% aqueous AlCl_3_. The solution was allowed to stand for 6 min at room temperature, then 2 mL 1M NaOH were added and the solution diluted with distilled water to 5 mL. It was then incubated for 15 min at room temperature. The absorbance of the solution was measured with a UV-Vis spectrophotometer at a wavelength of 510 nm using catechin as standard compound. Blank solutions were made by the same method, replacing the solvent with distilled water. Each test was replicated 3 times. The total flavonoid content was calculated using Eq. (2) and expressed as catechin equivalents (CE)

(2) $$\text{Total ​flavonoid ​content ​}=\text{ ​}\frac{\text{ ​ ​Catechin ​equivalent ​}\left(\text{CE}\right)\left(\text{mg}\right)}{\text{ ​g ​dry ​mass ​sample}}$$

### 2.6 Antioxidant activity

The antioxidant effect of the polyphenol extracts was measured using two different methods, namely the DPPH and FRAP. This was necessary since the DPPH method may not be able to accurately determine antioxidant properties of hydrophilic compounds due to solubility issues with DPPH. With FRAP, on the other hand, hydrophilic compounds can be accounted for (Ulewicz-Magulska and Wesolowski, 2019).

### 2.7 DPPH method

Based on Abozed et al. (2014) the DPPH test was conducted by mixing 90 μL of 0.15 mM DPPH (diphenyl-2-picrylhydrazyl) with 30 μL of test extract. The solution mixture was left in a dark room at room temperature for 30 min; subsequently its absorbance was measured at a wavelength of 515 nm. The control solution was made by mixing 4.9 mL of DPPH reagent with 0.1 mL of methanol and after 30 min in dark room at room temperature, the absorbance was measured at a wavelength of 515 nm. The antioxidant property of the sample was expressed as radical scavenging capacity (% inhibition, Eq. (3)) and as gallic acid equivalents (GAE, Eq. (4)).

(3) $$\text{\% ​Inhibition ​}=\text{ ​}\frac{\text{Control ​abs}-\text{sample ​abs}}{\text{Control ​abs}}\times 100\text{\%}$$

(4) $$\text{Antioxidant ​activity ​}=\text{ ​}\frac{\text{ ​Gallic ​acid ​equivalent ​}\left(\text{GAE}\right)\left(\text{μmol}\right)}{\text{ ​g ​dry ​mass ​sample ​}}$$

### 2.8 Ferric Reduction Ability of Plasma (FRAP)

Based on the procedure described by Benzie and Strain (1996), a sodium acetate buffer solution of pH 3.6 was prepared. 270 mg of TPTZ (2,4,6-tripyridyl-s-triazine) were dissolved in 50 mL 40 mM HCL. A 20 mM FeCl_3_.6H_2_O solution was prepared with distilled water. The FRAP reagent was made by mixing 25 mL of the acetate buffer, 2.5 mL of 10 mM TPTZ, and 2.5 mL of 20 mM FeCl_3_.6H_2_O solution. The volume was adjusted to 100 mL using distilled water.

For the assay, 60 μL sample were mixed with the FRAP reagent at a ratio of 1:1. The absorbance was measured at a wavelength of 593 nm. A standard solution was prepared using gallic acid and treated the same as a sample solution. Blank solutions were made by the same method with distilled water. All assays were repeated three times. The antioxidant activity was determined with Eq. (5) and expressed as gallic acid equivalents (GAE).

(5) $$\text{Antioxidant}\;\text{activity}=\frac{\text{Gallic}\;\text{acid}\;\text{equivalent}\;\left(\text{GAE}\right)\left(\text{μmol}\right)}{100\;\text{g}\;\text{dry}\;\text{mass}\;\text{sample ​}}$$

### 2.9 Statistical analysis

A randomized method was used to design the experiments where the first factor taken is the solvent of extraction, followed by the extraction method itself. All the experiments conducted in this study were repeated three times and the data reported are average values with standard deviations. All data in this study were analyzed with the Shapiro-Wilk normality test, One-Way ANOVA and a significance test with a value of α = 0.05. If the data are not normally distributed, a one-way ANOVA analysis cannot be applied. For non-normal data, the Kruskal-Wallis method was utilized, followed by a signification test using the Mann-Whitney test with a value of α = 0.1. Minitab 18 software was employed to conduct the analysis.

## 3 Results and discussion

### 3.1 Empty palm fruit bunches as raw material

Empty palm fruit bunches are the major by-product of the palm oil production. Besides cellulose, hemicellulose and lignin, it also contains polyphenolic compounds that can be extracted. Table 1 lists the results of maceration with three solvents. Maceration is the simplest extraction method to perform, though also the most time consuming one (Jeyaraj et al., 2021). In the Table 1, it was shown that there was a significant difference in term of the phenolics concentration in the extracts yielded from different methods and solvents.

**Table 1:** Total phenolic, flavonoid content, and antioxidant activity obtained after extraction of empty palm fruit bunches with different solvents (extraction method: maceration).

Extractives	70% Methanol	70% Ethanol	Dist. Water
Total Phenolic Content (mg GAE g^−1^ sample)	0.86^a^ ± 0.123	0.47^b^ ± 0.056	1.15^a^±0.195
Total Flavonoid Content (mg CE g^−1^ sample)	1.92^a^ ± 0.379	1.03^a^ ± 0.736	1.64^a^ ± 0.189
Antioxidant Activity DPPH (% Inhibition)	89.83^a^ ± 3.420	88.93^ab^ ± 0.990	82.66^b^ ± 3.120
Antioxidant Activity DPPH (μmol GAE g^−1^ sample)	4.045^a^ ± 0.424	3.828^a^ ± 0.210	3.117^a^ ± 0.879
Antioxidant Activity FRAP (μmol GAE g^−1^ sample)	19.02^a^ ± 0.717	12.54^b^ ± 0.415	10.98^b^ ± 3.830
Extract Yield (% w/w)	8.01^a^ ± 3.402	7.37^a^ ± 3.173	16.93^a^ ± 6.180

Note: All data in Table 1 are the average value of 3 independent experiments with standard deviations; letters in rows indicate a significant difference based on the results of the ANOVA One-Way test with a confidence level of 95%.

Distilled water and 70% methanol provided best total phenolic content, followed by ethanol. Although the total phenolic concentration results obtained here were still somewhat lower compared to those reported by Tsouko et al. (2019), they paralleled those reported by Ulewicz-Magulska and Wesolowski (2019). There it is stated that the use of 80% methanol and distilled water as solvents did not make a significant difference for the total concentration of phenolics in the extracts.

In the case of flavonoids, 70% methanol yielded the highest amount from the extract, but overall, the obtained data did not differ markedly. It would be expected that the total concentration of flavonoids in the extracts is lower than that of the phenolics since they are only a sub-group thereof. However, the standard for phenolics is gallic acid, while it is catechin for flavonoids. Thus, the two methods are not directly comparable. As a general rule, the more phenolics can be extracted, the more potent the antioxidant qualities of the extract should be. From Table 1 it is shown that 70% methanol as the solvent gave the highest result for the antioxidant activity determined by DPPH (%inhibition) and FRAP (μmol GAE/g sample), while water gave the lowest value of antioxidant activity determined by both methods. This could mean, that water extracted much more compounds (as indicated also by the yield obtained) that contributed false positive result for total phenolic determination. Moreover, some of the % inhibition values of DPPH obtained from our experiments were more than 85% present. This could indicate a plateau of the reaction and thus, conclusion should not be taken solely based on these DPPH % inhibition values, but also by looking at the other parameters.

We then also investigated different extraction methods (Maceration, Soxhlet, and Ultrasound Assisted Extraction/UAE) for 70% methanol as solvent. In Table 2, the results are depicted which were obtained with 2.5 g sample in 150 mL solvent. It can be seen that Soxhlet extraction tended to give slightly higher value for total phenolic and total flavonoid in comparison to the other methods. Also, Soxhlet Extraction with 70% methanol as solvent provided extract with the best antioxidant activity in comparison to other methods, as indicated by the DPPH (%inhibition) and FRAP value (μmol GAE/g sample).

**Table 2:** Total phenolic, flavonoids content, and antioxidant activity results using different extraction methods (solvent: 70% methanol).

Method	UAE	Soxhlet	Maceration
Total Phenolic Content (mg GAE g^−1^ sample)	0.60^b^ ± 0.057	0.80^a^ ± 0.052	0.71^ab^ ± 0.022
Total Flavonoid Content (mg CE g^−1^ sample)	2.98^b^ ± 0.575	3.97^a^ ± 0.328	3.57^ab^ ± 0.180
Antioxidant Activity DPPH (% Inhibition)	67.03^c^ ± 0.811	76.44^a^ ± 1.087	71.84^b^ ± 0.862
Antioxidant Activity DPPH (μmol GAE g^−1^ sample)	2.04^c^ ± 0.211	4.48^a^ ± 0.283	3.29^b^ ± 0.224
Antioxidant Activity FRAP (μmol GAE g^−1^ sample)	9.48^b^ ± 1.188	18.48^a^ ± 3.960	13.30^ab^ ± 1.672
Yield (% w/w)	8.47^a^ ± 2.083	15.34^a^ ± 3.307	5.72^ab^ ± 1.017

Note: All data in Table 2 are the average values of 3 independent samples with standard deviations; letters in rows indicate a significant difference based on the results of the ANOVA One-Way test with a confidence level of 95%.

It is possible that the elevated temperature during this extraction process increased the solubility of the metabolites (Nile et al., 2017). In general, Soxhlet extraction yielded the highest antioxidant activity compared to the other two methods. These results are in line with those of Khalil et al. (2018) and Nile et al. (2017), where the higher total phenolics resulted in higher antioxidant activity. However, compared to other studies found in literature, the data for empty fruit bunches of oil palms were still lower than for extracts of sugarcane pulp reported below.

The UAE method yielded the lowest value for all parameters. It is possible that the applied ultrasonic procedure was too mild and thus less effective for the rather dense biomass substrates to provide the needed turbulence and contact between solvent and extract (Wang and Weller, 2006). It is still necessary to research further the optimum power and duration of ultrasonication needed for the sufficient extraction.

### 3.2 Sugarcane pulp as resource for antioxidants

Waste from sugarcane production generally contains approximately 45–60% cellulose, 25% hemicellulose, 20–25% lignin and it is commonly used as a low-energy fuel or as fibrous reinforcement in composite materials (Hajiha and Sain, 2015). However, due to its low cost and high availability, sugarcane pulp can also serve as a worthwhile resource for phenolic compounds and its analysis of potential phenolic and flavone compounds as well as the chemical structures contained in sugarcane has been reported in Singh et al. (2015).

In Table 3 the results for maceration with alcohols and distilled water of sugarcane samples are displayed. The overall yield of extract mass was higher for sugarcane compared to extracts of empty palm fruit bunches (see Table 1 for comparison).

**Table 3:** Total phenolic, flavonoids, and antioxidant activity profile after extraction of sugarcane pulp as raw material with different solvents (extraction method: maceration).

Extractive	70% Methanol	70% Ethanol	Dist. Water
Total Phenolic Content (mg GAE g^−1^ sample)	1.35^b^ ± 0.096	1.67^a^ ± 0.097	0.8^c^ ± 0.037
Total Flavonoid Content (mg CE g^−1^ sample)	3.29^a^ ± 1.091	1.95^ab^ ± 0.605	0.50^b^ ± 0.236
Antioxidant Activity DPPH (% Inhibition)	85.67^a^ ± 1.080	86.02^a^ ± 0.911	78.45^b^ ± 0.632
Antioxidant Activity DPPH (μmol GAE g^−1^ sample)	1.67^a^ ± 0.116	1.82^a^ ± 0.123	1.12^b^ ± 0.477
Antioxidant Activity FRAP (μmol GAE g^−1^ sample)	30.81^a^ ± 1.950	15.45^c^ ± 1.080	25.09^b^ ± 1.189
Yield (% w/w)	12.05^a^ ± 2.102	10.00^a^ ± 3.128	19.85^a^ ± 3.275

Note: The data in Table 3 are the average value of three independent extractions with standard deviation; letters in rows indicate a significant difference based on the results of the ANOVA One-Way test with a confidence level of 95%.

No major differentiation, however, could be made between methanol and ethanol on total flavonoid content while there is difference on total phenolic content in the extracts obtained by three different solvents. Apparently, distilled water gave the lowest amount on both total phenolic and total flavonoid content.

With sugarcane, the highest total phenolics content could be extracted with 70% ethanol. This finding is rather similar with those of Venkatesan et al. (2019) on pine plant extraction. It seems that the phenolics in the sugarcane extract, like ferulic acid and coumaric acid have a similar polarity as 70% ethanol and thus go into solution easily under these experimental conditions (Zhao et al., 2015).

In Table 3, we can see that some of the % inhibition values of DPPH obtained from our experiments were more than 85% present. This could indicate a plateau of the reaction and thus, conclusion should not be taken solely based on these DPPH % inhibition values, but also by looking at the other parameters. While the values of antioxidant activity resulted from DPPH assay are rather similar for all extracts, the highest antioxidant activity was shown by the extract from 70% methanol as measured with FRAP method. This data trend somewhat aligns well the flavonoid content data using catechin as standard compound, which also in concordance with the work of Vianney et al. (2018) and Vijayalaxmi et al. (2015), reporting that methanol seems to be well-suited as solvent to yield flavonoids, while ethanol and water dissolved less flavonoids. There is a possibility that the antioxidant activity of sugarcane methanol extract is mostly contributed from the extracted flavonoid compounds.

Table 4 presents a comparison of extraction methods for sugarcane pulp by means of 70% methanol. It is obvious that using Soxhlet method, the highest yield could be obtained, while UAE gave the least extract yield. As also observed with empty fruit bunches of palm oil, Soxhlet extraction provided the highest amount of phenolics and flavonoids.

**Table 4:** Impact of the extraction method on total phenolic and flavonoids concentration as well as antioxidant activity for sugarcane pulp (solvent: 70% methanol).

Method	UAE	Soxhlet	Maceration
Total Phenolic Content (mg GAE g^−1^ sample)	0.30^c^ ± 0.007	0.96^a^ ± 0.003	0.41^b^ ± 0.008
Total Flavonoid Content (mg CE g^−1^ sample)	2.28^c^ ± 0.104	5.48^a^ ± 0.202	4.47^b^ ± 0.180
Antioxidant Activity DPPH (% Inhibition)	84.29^a^ ± 0.611	71.03^c^ ± 0.316	74.76^b^ ± 0.593
Antioxidant Activity DPPH (μmol GAE g^−1^ sample)	1.60^a^ ± 0.019	0.75^c^ ± 0.103	0.93^b^ ± 0.021
Antioxidant Activity FRAP (μmol GAE g^−1^ sample)	11.69^c^ ± 1.471	36.20^a^ ± 0.849	21.49^b^ ± 1.531
Yield (% w/w)	8.17^b^ ± 2.036	19.00^a^ ± 2.271	9.77^b^ ± 3.073

Note: The data in Table 4 are the average values with standard deviations of three independent experiments; letters in rows indicate a significant difference based on the results of the ANOVA One-Way test with a confidence level of 95%.

While the data of DPPH assay is rather inconclusive, we can see in Table 4 that the FRAP assay data is in line with value of phenolic and flavonoid content. As has been expected, the extract from Soxhlet extraction gave the highest antioxidant activity as measured by FRAP method (μmol GAE g^−1^ sample).

Although some researchers like Juttuporn et al. (2017) proposed UAE as the potential best technique for industrial application for the sake of shorter extraction time, this report rather complements other reports that still indicate the older Soxhlet extraction as the method with the highest extraction yield. It seems that the heating process in Soxhlet extraction provided a greater disruption of the plant cell wall, a greater penetration of the solvent, and a greater release of extractable compounds. There are of course still possibilities to optimize the UAE condition (power, time, solvent choice) to achieve higher extraction result.

## 4 Conclusion

Comparative research was performed to investigate feasible extraction methods of fibrous waste from agriculture, i.e. empty fruit bunches from palm oil production and sugarcane pulp. Those agricultural waste are highly available and plentiful in Indonesia and are currently destroyed by burning, causing air pollution. Methods were studied that would allow extraction of the polyphenols in a simple and economical manner, so it may feasibly be adopted by the corresponding potential small to medium enterprise/industry.

The best extraction results for palm bunches and sugarcane pulp were obtained by the Soxhlet method at 60 °C for 2 h with 70% methanol as the solvent, resulted in about two times higher values for total phenolic content, flavonoid content and FRAP antioxidant activity as well as extract mass (yield) in comparison to maceration and Ultrasonic Assisted Extraction (UAE) methods with the conditions as applied in this work. In future research, extraction conditions still need to be further optimized in terms of temperature and duration of extraction, perhaps also concerning the sample mass to solvent volume ratio.

## Declarations

### Author contribution statement

Leonardo Satriono Putra: Performed the experiments; Wrote the paper.

Johan Sukweenadhi: Analyzed and interpreted the data; Contributed reagents, materials, analysis tools or data.

Clairine Nathania, Enrico Setiawan Wibowo: Performed the experiments; Analyzed and interpreted the data.

Gisela Buschle-Diller: Analyzed and interpreted the data; Wrote the paper.

Maria Goretti Marianti Purwanto: Conceived and designed the experiments; Analyzed and interpreted the data; Wrote the paper.

### Funding statement

This work was supported by 10.13039/501100010447Kementerian Riset, Teknologi dan Pendidikan Tinggi Indonesia (PDUPT 2021).

### Data availability statement

Data included in article/supplementary material/referenced in article.

### Declaration of interests statement

The authors declare no conflict of interest.

### Additional information

No additional information is available for this paper.
